# Nitrogen Kinetic Isotope Effects of Nitrification by the Complete Ammonia Oxidizer Nitrospira inopinata

**DOI:** 10.1128/mSphere.00634-21

**Published:** 2021-12-08

**Authors:** Shurong Liu, Man-Young Jung, Shasha Zhang, Michael Wagner, Holger Daims, Wolfgang Wanek

**Affiliations:** a University of Vienna, Centre for Microbiology and Environmental Systems Science, Vienna, Austria; b University of Vienna, The Comammox Research Platform, Vienna, Austria; c Shenzhen Campus of Sun Yat-Sen University, School of Agriculture, Shenzhen, China; d Department of Biology Education, Jeju National University, Jeju, Republic of Korea; e Interdisciplinary Graduate Program in Advance Convergence Technology and Science, Jeju National University, Jeju, Republic of Korea; f Aalborg University, Department of Chemistry and Bioscience, Aalborg, Denmark; University of California, Davis

**Keywords:** comammox, isotope fractionation, kinetic isotope effect, nitrification

## Abstract

Analysis of nitrogen isotope fractionation effects is useful for tracing biogeochemical nitrogen cycle processes. Nitrification can cause large nitrogen isotope effects through the enzymatic oxidation of ammonia (NH_3_) via nitrite (NO_2_^−^) to nitrate (NO_3_^−^) (^15^ε_NH4+→NO2-_ and ^15^ε_NO2-→NO3-_). The isotope effects of ammonia-oxidizing bacteria (AOB) and archaea (AOA) and of nitrite-oxidizing bacteria (NOB) have been analyzed previously. Here, we studied the nitrogen isotope effects of the complete ammonia oxidizer (comammox) Nitrospira inopinata that oxidizes NH_3_ to NO_3_^−^. At high ammonium (NH_4_^+^) availability (1 mM) and pH between 6.5 and 8.5, its ^15^ε_NH4+→NO2-_ ranged from −33.1 to −27.1‰ based on substrate consumption (residual substrate isotopic composition) and −35.5 to −31.2‰ based on product formation (cumulative product isotopic composition), while the ^15^ε_NO2-→NO3-_ ranged from 6.5 to 11.1‰ based on substrate consumption. These values resemble isotope effects of AOB and AOA and of NOB in the genus *Nitrospira*, suggesting the absence of fundamental mechanistic differences between key enzymes for ammonia and nitrite oxidation in comammox and canonical nitrifiers. However, ambient pH and initial NH_4_^+^ concentrations influenced the isotope effects in *N. inopinata*. The ^15^ε_NH4+→NO2-_ based on product formation was smaller at pH 6.5 (−31.2‰) compared to pH 7.5 (−35.5‰) and pH 8.5 (−34.9‰), while ^15^ε_NO2-→NO3-_ was smaller at pH 8.5 (6.5‰) compared to pH 7.5 (8.8‰) and pH 6.5 (11.1‰). Isotopic fractionation via ^15^ε_NH4+→NO2-_ and ^15^ε_NO2-→NO3-_ was smaller at 0.1 mM NH_4_^+^ compared to 0.5 to 1.0 mM NH_4_^+^. Environmental factors, such as pH and NH_4_^+^ availability, therefore need to be considered when using isotope effects in ^15^N isotope fractionation models of nitrification.

**IMPORTANCE** Nitrification is an important nitrogen cycle process in terrestrial and aquatic environments. The discovery of comammox has changed the view that canonical AOA, AOB, and NOB are the only chemolithoautotrophic organisms catalyzing nitrification. However, the contribution of comammox to nitrification in environmental and technical systems is far from being completely understood. This study revealed that, despite a phylogenetically distinct enzymatic repertoire for ammonia oxidation, nitrogen isotope effects of ^15^ε_NH4+→NO2-_ and ^15^ε_NO2-→NO3-_ in comammox do not differ significantly from those of canonical nitrifiers. Thus, nitrogen isotope effects are not suitable indicators to decipher the contribution of comammox to nitrification in environmental samples. Moreover, this is the first systematic study showing that the ambient pH and NH_4_^+^ concentration influence the isotope effects of nitrifiers. Hence, these key parameters should be considered in comparative analyses of isotope effects of nitrifiers across different growth conditions and environmental samples.

## INTRODUCTION

Natural abundance isotope techniques have proven useful for studying nitrogen (N) transformation processes in aquatic and terrestrial ecosystems (1–3). Several process-oriented models that integrate isotope effects have been reported (4, 5). However, the successful integration of N isotopic composition into N cycle models requires the knowledge of the accurate isotope effects of each N transformation process. Until now, multiple N isotope effects have been reported based on soil and groundwater studies and for microbial isolates (3).

Nitrification represents a two-step N cycle process where ammonia (NH_3_) is first oxidized to nitrite (NO_2_^−^) by ammonia-oxidizing bacteria (AOB) and archaea (AOA), followed by the oxidation of NO_2_^−^ to nitrate (NO_3_^−^) by nitrite-oxidizing bacteria (NOB). Intriguingly, nitrification has shown much larger isotope effects than other N cycle processes, including biological N_2_ fixation, mineral N uptake, ammonification, and denitrification (3). Isotope effects (^15^ε_NH4+→NO2-_) for ammonia oxidation to NO_2_^−^ have been determined in enriched and pure cultures of AOB and AOA (6–9). The AOB strain Nitrosomonas europaea exhibited an isotope effect of −38 to –32‰, whereas Nitrosomonas marina, *Nitrosomonas* sp. C-113a, and Nitrosospira tenuis showed smaller isotope effects in the range of −25 to −14‰ (6, 7). Measured isotope effects of AOA were in the same range as those of AOB, with a large variation of −21‰ ± 10‰ (8), regardless of whether the AOA had been cultured from marine or geothermal sources (9). The factors causing the large variations of the measured isotope effects in AOA and AOB have remained largely unknown. Possible causes include differences in the pH and in initial NH_4_^+^ concentrations between studies, amino acid substitutions in ammonia monooxygenase (AMO; the key enzyme for ammonia oxidation in AOB and AOA), and any isotope effect possibly involved in the subsequent oxidation of the AMO product hydroxylamine (NH_2_OH) to NO_2_^−^, and with gaseous N losses via nitric oxide (NO) or nitrous oxide (N_2_O) (7, 9).

Only a few studies have addressed isotopic fractionation during NO_2_^−^ oxidation. The isotope effect of the key nitrite-oxidizing enzyme, nitrite oxidoreductase (NXR) (^15^ε_NO2→NO3-_), became a focus of attention when the difference of δ^15^N between NO_3_^−^ and NO_2_^−^ in marine samples turned out to be surprisingly large (10, 11). This was unexpected, because processes consuming NO_2_^−^ (such as NO_2_^−^ oxidation) or NO_3_^−^ (denitrification) should increase the δ^15^ N residual NO_2_^−^ and NO_3_^−^, respectively, and lead to a smaller offset in the δ^15^N than observed. Hence, these results were taken as an indication for a process that actually decreased the δ^15^NO_2_^−^ relative to δ^15^NO_3_^−^ (12). Indeed, a subsequent study revealed an inverse isotope effect of NXR, where the substrate (NO_2_^−^) became more depleted in ^15^N compared to the product (NO_3_^−^) in the marine NOB Nitrococcus mobilis (10). This contrasts with most other enzymatic processes of the N cycle, where the substrate becomes ^15^N enriched and the product ^15^N depleted. Three explanations for such inverse kinetic isotope effects were studied, i.e., equilibrium isotope effects between NO_2_^−^ and nitrous acid before reaction, reaction reversibility at the enzyme level, and real inverse kinetic isotope fractionation (10). The inverse isotope effect of NXR most likely originates at the enzyme level, where larger force constants in the transition state explain the inverse kinetic isotope effect when stretching vibrational contributions dominate the kinetic isotope effect (13). Follow-up studies demonstrated inverse isotope effects of the NOB *Nitrococcus* and *Nitrobacter* to be similar and around 20.5‰, whereas the inverse isotope effects of the NOB *Nitrospira* and *Nitrospina* were less pronounced and close to 9.5‰ (11, 14), and those of anammox bacteria were larger with 30.1 to 45.3‰ (15). The cause for the quantitatively different isotope effects of ^15^ε_NO2-→NO3-_ in the diverse NOB remained unclear. Possibly responsible factors include the orientation of the membrane-attached NXR toward either the cytoplasm or periplasm, kinetic differences among the known forms of NXR, and the reversibility of NO_2_^−^ oxidation by this enzyme (i.e., its capability to also catalyze NO_3_^−^ reduction) (14).

Complete ammonia oxidizers (comammox organisms), which oxidize NH_3_ to NO_3_^−^ on their own, were recently discovered in the genus *Nitrospira* (16, 17). Comammox organisms are widespread in natural and engineered ecosystems (18). They possess a distinct form of AMO, which is phylogenetically moderately related to the AMO of betaproteobacterial AOB (16, 17). Generally, it is assumed that NH_3_ and not NH_4_^+^ is the substrate for AMO in AOB, AOA, and comammox (19, 20). The only available comammox isolate, Nitrospira inopinata, has a very high substrate affinity for ammonia that exceeds the affinities of all characterized AOB and several AOA (21). Comammox bacteria share a highly similar NXR with canonical (only NO_2_-oxidizing) *Nitrospira*, but the affinity of N. inopinata for NO_2_^−^ is much lower than that reported for canonical *Nitrospira* (21, 22). The unique kinetic properties of comammox, and the distinct AMO, raise the question of whether NH_3_ and NO_2_^−^ oxidation by comammox might show comparable or different isotope effects than found in the canonical nitrifiers. So far, however, no isotope fractionation data from comammox have been available. In this study, we analyzed the N kinetic isotope effects of ammonia oxidation (^15^ε_NH4+→NO2-_) and nitrite oxidation (^15^ε_NO2-→NO3-_) of a pure culture of *N. inopinata*. In addition, we explored whether the isotope effect is influenced by selected environmental factors (medium pH and initial NH_4_^+^ concentration). The results provide important constraints for the interpretation of natural abundance stable isotope ratios for N compounds in systems where comammox *Nitrospira* are prevalent.

## RESULTS

### Experiment 1: ammonia oxidation with an initial concentration of 1 mM NH_4_*^+^*.

**FIG 1:**
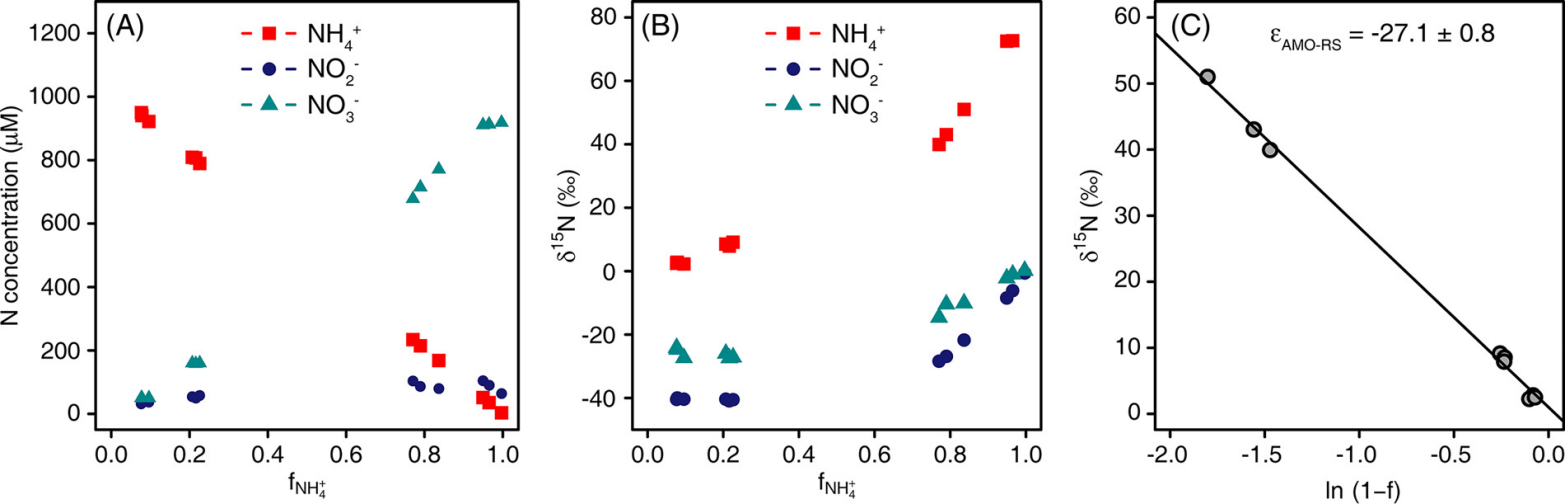
Kinetic isotope effect of *N. inopinata* cultivated in CaCO_3_-buffered medium with 1 mM ammonium (NH_4_^+^) initial concentration. (A) Concentrations of NH_4_^+^, nitrite (NO_2_^−^), and nitrate (NO_3_^−^). (B) Isotopic signatures of NH_4_^+^, NO_2_^−^, and NO_3_^−^. (C) ^15^ε_NH4+→NO2-_ based on the residual substrate (ε_AMO-RS_).

Within 2 weeks of this incubation experiment, N. inopinata oxidized the initially provided 1 mM NH_4_^+^ to approximately 90% NO_3_^−^ and 10% NO_2_^−^ (Fig. 1A). A transient accumulation of NO_2_^−^ was also observed in previous studies with *N. inopinata*, where the residual NO_2_^−^ was finally converted to NO_3_^−^ during prolonged incubations after NH_4_^+^ depletion (16, 21). The ratio of NH_4_^+^ consumption to NO_2_^−^ plus NO_3_^−^ formation was close to 1.0, indicating that ammonia was almost stoichiometrically oxidized to NO_2_^−^ and NO_3_^−^ (Fig. 1A). The initial δ^15^N of NH_4_^+^ was −0.6‰. The δ^15^N of NH_4_^+^ increased exponentially with incubation time, along with an increase of δ^15^N of NO_2_^−^ and NO_3_^−^. Moreover, the δ^15^N of NO_2_^−^ was depleted compared to the δ^15^N of NH_4_^+^ and NO_3_^−^ during the incubation (Fig. 1B). The ^15^ε_NH4+→NO2-_ was −27.1‰ ± 0.8‰ based on the residual substrate (equation 2, Fig. 1C, and Table 1) and −32.2‰ ± 1.4‰ based on the cumulative product (Table 1), which was in agreement with the ^15^ε_NH4+→NO2-_ (−38 to −14‰) of canonical AOB and AOA (7, 8). The ^15^ε_NO2-→NO3-_ was 7.6‰ ± 0.2‰ based on the residual substrate from the Solver model (Table 1), which was close to the canonical *Nitrospira* NOB (9‰) (11, 14).

**TABLE 1:** Modeled kinetic isotope effects (mean ± s.d., *n* = 3) of AMO and NXR of *Nitrospira inopinata*^*a*^

NH_4_^+^ (mM)	pH	NH_4_^+^/NO_2_^−^ oxidation rate (μM/h)	NH_4_^+^ oxidation rate [μmol N (mg protein)^−1^ h^−1^]	ε_AMO-RS_ (‰)	ε_AMO-CP_ (‰)	ε_NXR-RS_ (‰)
1	6.5	15.8 ± 2.6	*V*_max_ (12.8)	−30.1 ± 0.5	−31.2 ± 0.3	11.1 ± 0.6
1	7.5	18.6 ± 0.7	*V*_max_ (12.8)	−31.6 ± 0.5	−35.5 ± 0.2	8.8 ± 0.6
1	8.5	14.0 ± 1.7	*V*_max_ (12.8)	−33.1 ± 0.8	−34.9 ± 1.6	6.5 ± 1.0
0.1	8.2	5.8 ± 0.3^*b*^	2.1 ± 0.1	−19.7	−17.3	6.2
0.25	8.2	8.6 ± 0.4	3.1 ± 0.1	−21.1 ± 1.3	−21.2 ± 2.3	10.8 ± 1.1
0.5	8.2	10.7 ± 0.5	3.8 ± 0.2	−24.8 ± 0.2	−24.3 ± 1.9	10.5 ± 2.3
1	8.2	6.2 ± 0.4^*c*^		−27.1 ± 0.8	−32.2 ± 1.4	7.6 ± 0.2
1	8.2	39.5 ± 4.6				9.2 ± 0.5

a Modeled kinetic isotope effects (mean ± standard deviation, *n* = 3) of AMO and NXR of *Nitrospira inopinata* based on the Solver model at pH 6.5 to 8.5 with initial NH_4_^+^ concentrations of 0.1 to 1 mM.

b AOA/AOB medium buffered with CaCO_3_ (pH around 8.2) was used for this batch experiment with 0.1, 0.25, and 0.5 mM NH_4_^+^.

c The batch experiment was performed with CaCO_3_-buffered medium but with much less biomass.

### Experiment 2: nitrite oxidation with an initial concentration of 1 mM NO_2_.

**FIG 2:**
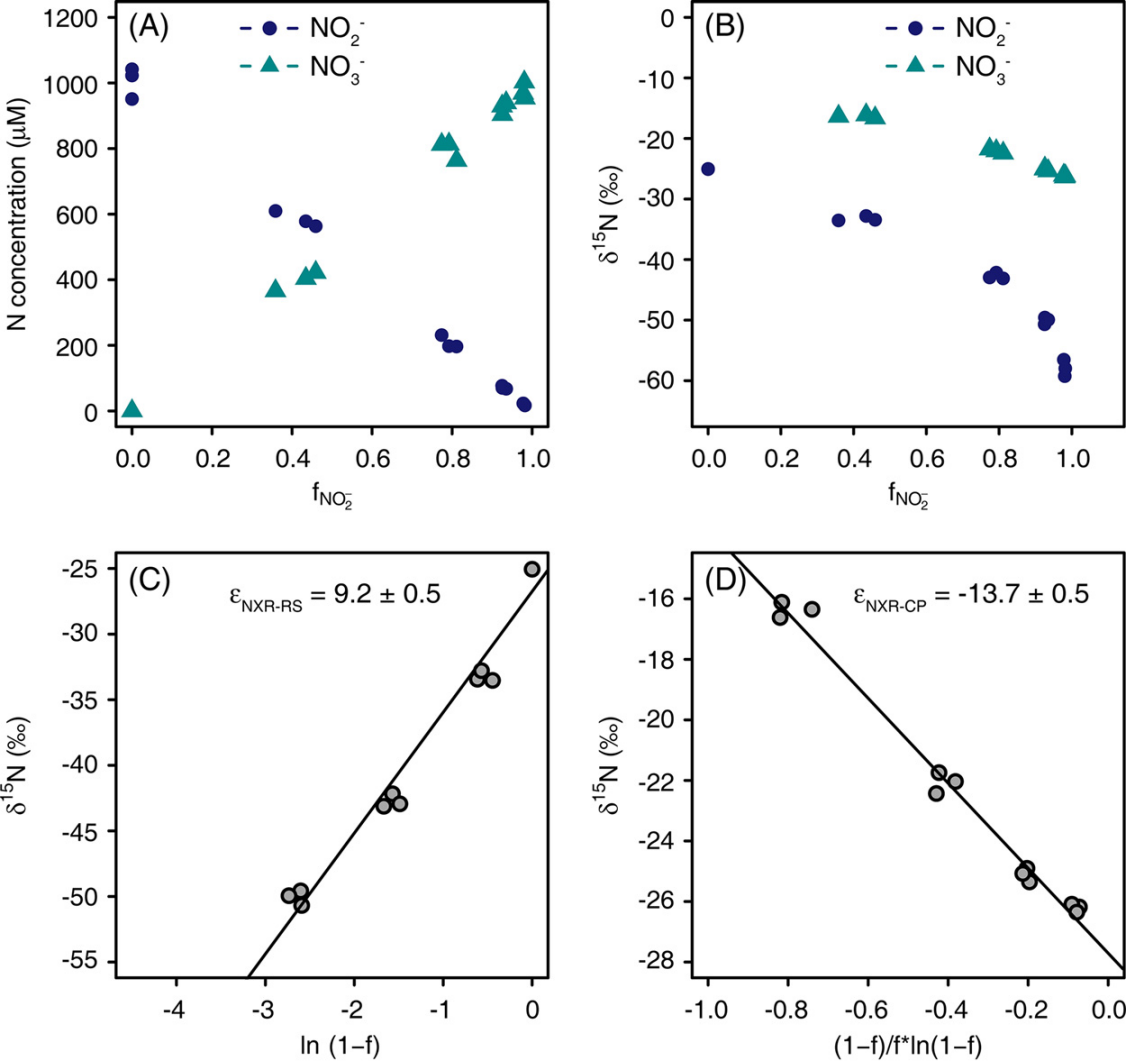
Kinetic isotope effect of *N. inopinata* cultivated in CaCO_3_-buffered medium with 1 mM NO_2_^−^ initial concentration. (A) Concentrations of NO_2_^−^ and NO_3_^−^. (B) Isotopic signatures of NO_2_^−^ and NO_3_^−^. (C and D) ^15^ε_NO2-→NO3-_ based on the residual substrate (ε_NXR-RS_) (C) and cumulative product (ε_NXR-CP_) (D).

In a subsequent experiment, the ^15^ε_NO2-→NO3-_ was directly measured from a batch incubation with NO_2_^−^ as a substrate. Since *N. inopinata* is unable to utilize NO_2_^−^ as an N source for assimilation (16), growth was not expected to occur during the incubation experiment with NO_2_^−^. Thus, we used an already highly concentrated cell suspension to analyze the ^15^ε_NO2-→NO3-_. Within 2 days of incubation, the initially provided 1 mM NO_2_^−^ was almost stoichiometrically oxidized to NO_3_^−^ (Fig. 2A). The ratio of NO_2_^−^ oxidation to NO_3_^−^ production was 1.03 ± 0.03. The initial δ^15^N of NO_2_^−^ was −25‰, and the δ^15^N of both NO_2_^−^ and NO_3_^−^ decreased along with NO_2_^−^ oxidation. In agreement with previous studies of canonical NOB (see introduction), the δ^15^N of NO_2_^−^ was depleted compared to the δ^15^N of NO_3_^−^ during NO_2_^−^ oxidation (Fig. 2B). The calculated, inverse isotope effect of ^15^ε_NO2-→NO3-_ was 9.2‰ ± 0.5‰ based on the Rayleigh models for the residual substrate (Fig. 2C), which was similar with the above-mentioned ^15^ε_NO2-→NO3-_ (7.6‰ ± 0.2‰) calculated from the Solver model during NH_3_ oxidation. Figure 2D shows the calculated ^15^ε_NO2-→NO3-_ based on the cumulative product.

### Experiment 3: ammonia oxidation with an initial concentration of 0.1, 0.25, and 0.5 mM NH_4_*^+^*.

**FIG 3:**
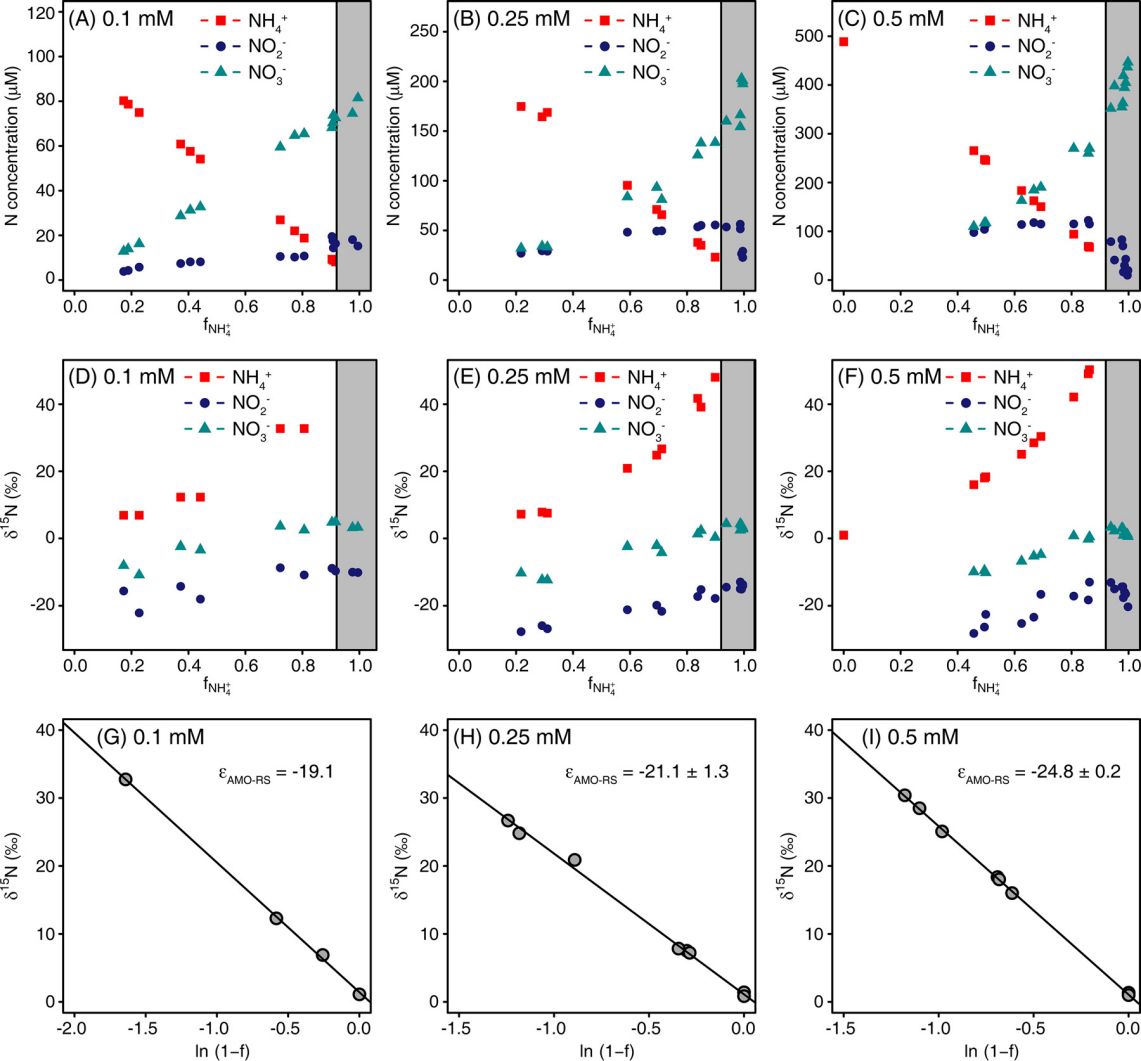
Kinetic isotope effect of *N. inopinata* cultivated in CaCO_3_-buffered medium with 0.1, 0.25, and 0.5 mM NH_4_^+^ initial concentration. (A to C) Concentrations of NH_4_^+^, NO_2_^−^, and NO_3_^−^. (D to F) Isotopic signatures of NH_4_^+^, NO_2_^−^, and NO_3_^−^. (G to I) ^15^ε_NH4+→NO2-_ based on the residual substrate (ε_AMO-RS_).

Similar patterns of NH_4_^+^ oxidation, NO_2_^−^ production and consumption, and NO_3_^−^ production were observed for all tested initial NH_4_^+^ concentrations (Fig. 3A to C). However, the NH_4_^+^ oxidation rates increased with higher initial NH_4_*^+^* concentrations (Table 1). The concentration of transiently accumulated NO_2_^−^ also increased with the initial NH_4_^+^ concentration (Fig. 3A to C). The δ^15^N of NH_4_^+^ increased with ongoing NH_4_^+^ oxidation, along with an increase in the δ^15^N of NO_2_^−^ and NO_3_^−^. After about 93% of the NH_4_^+^ had been consumed, a pronounced decrease in the δ^15^N of NO_2_^−^ was observed, which was consistent with the net consumption of NO_2_^−^ (Fig. 3, especially Fig. 3D to F). The ^15^ε_NH4+→NO2-_ values based on the residual substrate were significantly (*P* < 0.05) larger for the 0.5 mM initial NH_4_^+^ concentration (−24.8‰) compared to that for the 0.25 mM initial NH_4_^+^ concentration (−21.1‰). The ^15^ε_NH4+→NO2-_ was smallest (−19.1‰) for the 0.1 mM initial NH_4_^+^ concentration among all the tested initial NH_4_^+^ concentrations (Fig. 3G to I). The calculated ^15^ε_NH4+→NO2-_ values based on the cumulative product were similar to those based on the residual substrate, with values of −17.3, −21.2, and −24.3‰ for the 0.1, 0.25, and 0.5 mM NH_4_^+^ addition, respectively (Table 1). As outlined below (see Discussion), the weaker ^15^ε_NH4+→NO2-_ at the lowest NH_4_^+^ concentration could be due to the low NH_4_^+^ oxidation rates with 0.1 mM NH_4_^+^ [2.1 μmol N (mg protein)^−1^ h^−1^; total protein content was ∼2.8 μg ml^−1^], which was significantly smaller than the *V*_max_ [12.8 μmol N (mg protein)^−1^ h^−1^] of *N. inopinata* (21). The ^15^ε_NO2-→NO3-_ values based on the Solver model were 6.2, 10.8, and 10.5‰ for the 0.1, 0.25, and 0.5 mM NH_4_^+^ treatments, respectively (Table 1). No significant difference of ^15^ε_NO2-→NO3-_ was observed between the 0.25 and 0.5 mM initial NH_4_^+^ concentration treatments.

### Experiment 4: ammonia oxidation at different pH values with an initial concentration of 1 mM NH_4_*^+^*.

**FIG 4:**
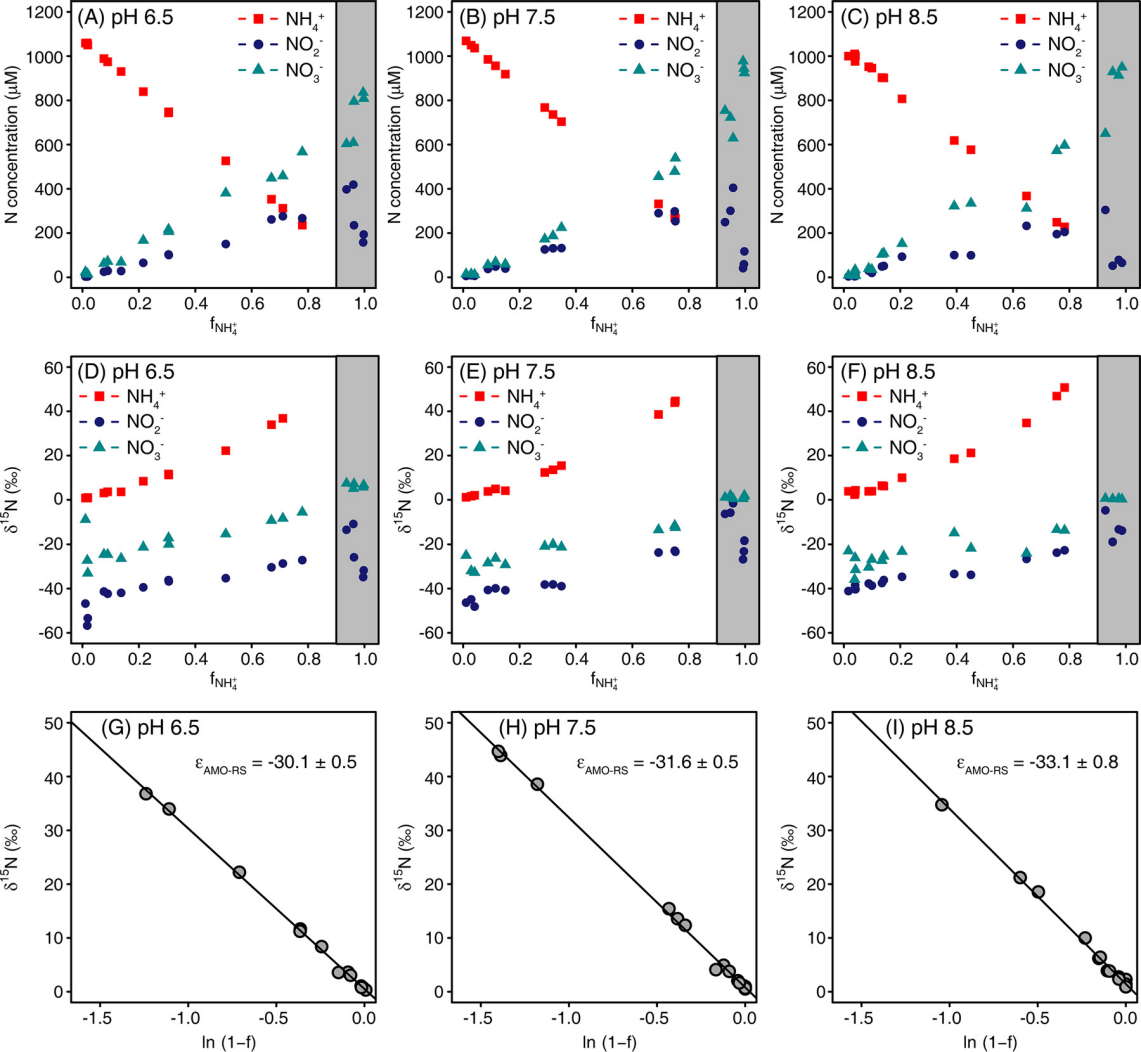
Kinetic isotope effect of *N. inopinata* cultivated with 1 mM NH_4_^+^ (initial concentration) at pH 6.5, 7.5, and 8.5. (A to C) Concentrations of NH_4_^+^, NO_2_^−^ and NO_3_^−^. (D to F) Isotopic signatures of NH_4_^+^, NO_2_^−^, and NO_3_^−^. (G to I) ^15^ε_NH4+→NO2-_ based on the residual substrate (ε_AMO-RS_).

In this experiment, the maximum NH_4_^+^ oxidation rates (day 3 to 4 for pH 6.5 and 7.5; day 4 to 5 for pH 8.5) were significantly (*P* < 0.05) lower at pH 8.5 than at pH 7.5 (Table 1 and Fig. 4A to C). The NO_2_^−^ concentration was significantly (*P* < 0.05) higher at pH 6.5 (361 μM) than that at pH 8.5 (234 μM), which was consistent with the trend of maximum NO_2_^−^ oxidation rate (this was calculated during the period of NO_2_^−^ oxidation when NH_4_^+^ was almost completely consumed) that was significantly (*P* < 0.05) lower at pH 6.5 (9.7 μM h^−1^) than at pH 8.5 (13.5 μM h^−1^). The δ^15^N of NH_4_^+^, NO_2_^−^, and NO_3_^−^ showed similar patterns as in the other experiments, i.e., the δ^15^N of NH_4_^+^, NO_2_^−^, and NO_3_^−^ increased simultaneously with ammonia oxidation until more than 90% NH_4_^+^ was oxidized, followed by a decrease in the δ^15^N of NO_2_^−^ until the end of the incubations (Fig. 4D to F). There was no significant difference for the ^15^ε_NH4+→NO2-_ calculated based on the residual substrate among the three pH levels (Table 1 and Fig. 4G to I), but the ^15^ε_NH4+→NO2-_ based on product formation was significantly (*P* < 0.05) weaker at pH 6.5 than at pH 7.5 and pH 8.5 (Table 1). Interestingly, the δ^15^N of NO_2_^−^ was significantly (*P* < 0.05) lower at pH 6.5 than at pH 8.5 at the beginning of the experiment. Moreover, the pH affected the isotope effect of NO_2_^−^ oxidation significantly, where the ^15^ε_NO2-→NO3-_ was significantly (*P* < 0.05) weaker at pH 8.5 than that at pH 7.5 and 6.5, based on the Solver model (Table 1).

## DISCUSSION

### Isotope effects of ammonia and nitrite oxidation by *N*. *inopinata*.

The measured N isotope effect for ammonia oxidation (^15^ε_NH4+→NO2-_) by *N*. *inopinata* (residual substrate [RS], −33.0 to −30.7‰; cumulative product [CP], −35.5 to −31.2‰) with an initial substrate concentration of 1 mM NH_4_^+^ fell into the range of ^15^ε_NH4+→NO2-_ values determined previously for AOB (−38.2 to −14.2‰) and AOA (−41 to −13‰) (7–9) (Table 2), as well as the measured N isotope effect of the two AOA species Nitrososphaera gargensis (RS, −22.3‰; CP, −32.8‰) and Nitrosocosmicus oleophilus that were also determined in this study (RS, −36.1‰; CP, −36.3‰) (see Fig. S1 and S2 in the supplemental material). However, isotope fractionation data are currently still lacking for many phylogenetic lineages of AOB and AOA. The most similar isotope effects, compared with *N. inopinata*, have been reported for Nitrosomonas europaea (−38.2‰) and Nitrosomonas eutropha (−32.8‰) (6, 7) (Table 2). In this context, it is noteworthy that comammox *Nitrospira*, betaproteobacterial AOB, and AOA possess three phylogenetically different types of AMO (16, 17, 23). Moreover, the characterized comammox *Nitrospira* and many AOA have a much higher substrate affinity for NH_3_ than AOB (21, 24, 25). Despite the distinct phylogenetic and kinetic properties of the AMO forms, no difference in the magnitude of ^15^ε_NH4+→NO2-_ between comammox, AOB, and AOA became apparent. This result may indicate that the enzymatic mechanism and transition states of the NH_3_ oxidation step catalyzed by AMO are similar across all ammonia oxidizers. However, the reported ^15^ε_NH4+→NO2-_ values varied strongly, even within one species or strain, in previous research (7–9). Hence, the kinetic isotope effects of ammonia oxidizers may be modulated by environmental factors, some of which have been investigated in our study (see below).

**TABLE 2:** Compilation of kinetic isotope effects of canonical AOA, AOB, and NOB

Nitrifier group	Strain	Initial substrate (mM)	pH	ε_RS_ (‰)	ε_CP_ (‰)	Reference
AOA	Nitrosopumilus adriaticus	1	7.6	−32 ± 1	−40 ± 1	Mooshammer et al. (33)
	Nitrososphaera viennensis	1−2	7.5	−32 ± 1	−39	Mooshammer et al. (33)

	Nitrososphaera gargensis	0.25	8.2	−22 ± 0	−33 ± 2	This study (Fig. S1 and S2)
	Nitrosocosmicus oleophilus	1	7.5	−36 ± 5	−36 ± 5	This study (Fig. S1 and S2)

	AOA enrichment CN25				−22 ± 5	Santoro and Casciotti (8)
	AOA enrichment CN75	0.01−0.075			−21 ± 10	Santoro and Casciotti (8)
	AOA enrichment CN150				−22 ± 5	Santoro and Casciotti (8)

	“Candidatus *Nitrosocaldus*”	0.2	8.2−8.6		−25 ± 2	Nishizawa et al. (9)
	“Candidatus *Nitrosocaldus*”	14	8.0		−32 ± 1	Nishizawa et al. (9)

AOB	Nitrosomonas europaea	4.7−25	7.5	−35 ± 3	−32 ± 6	Mariotti et al. (6)
	Nitrosomonas europaea	38			−32 to −25	Yoshida (30)

	Nitrosomonas europaea	1			−38 ± 2	Casciotti et al. (7)
	Nitrosomonas marina	2			−14 ± 4	Casciotti et al. (7)
	*Nitrosomonas* sp. C-113a	2	8.0		−19 ± 1	Casciotti et al. (7)
	Nitrosospira tenuis	1			−25 ± 1	Casciotti et al. (7)
	Nitrosomonas eutropha	1			−33 ± 2	Casciotti et al. (7)

	*Nitrosomonas* sp. C-113a					Casciotti et al. (43)
	Nitrosococcus oceani	0.005−0.05	8.2		−46 to −30	Casciotti et al. (43)
	Nitrosospira briensis					Casciotti et al. (43)

NOB	Nitrococcus mobilis			20 ± 3		Buchwald and Casciotti (11)
	*Nitrobacter* sp. Nb 355	0.05	8.2	21 ± 3		Buchwald and Casciotti (11)
	Nitrospira marina			9 ± 2		Buchwald and Casciotti (11)

	*Nitrospira* sp. Ecomares 2.1	0.5−1	7.5	10 ± 1		Jacob et al. (14)
	*Nitrospina watsonii* 347	0.6−1.6		10 ± 1		Jacob et al. (14)

	Nitrospira moscoviensis	1	7.5	9 ± 1		This study (Fig. S3)

10.1128/mSphere.00634-21.1 FIG S1 Ammonia oxidation (A), isotopic signature (B) of NH_4_^+^ and NO_2_^−^, and ^15^ε_NH4+→NO2-_ based on substrate (ε_AMO-RS_) (C) and product (ε_AMO-CP_) (D) of Nitrososphaera gargensis cultivated in CaCO_3_-buffered medium with 0.25 mM NH_4_^+^ addition. Download FIG S1, TIF file, 0.06 MB. Copyright © 2021 Liu et al. 2021 Liu et al. https://creativecommons.org/licenses/by/4.0/ This content is distributed under the terms of the Creative Commons Attribution 4.0 International license.

10.1128/mSphere.00634-21.2 FIG S2 Ammonia oxidation (A), isotopic signature (B) of NH_4_^+^ and NO_2_^−^, and ^15^ε_NH4+→NO2-_ based on substrate (ε_AMO-RS_) (C) and product (ε_AMO-CP_) (D) of Nitrosocosmicus oleophilus with 1 mM NH_4_^+^ addition. Download FIG S2, TIF file, 0.07 MB. Copyright © 2021 Liu et al. 2021 Liu et al. https://creativecommons.org/licenses/by/4.0/ This content is distributed under the terms of the Creative Commons Attribution 4.0 International license.

Like canonical NOB (10, 11, 14), *N. inopinata* displayed an inverse ^15^ε_NO2-→NO3-_, meaning that ^15^NO_2_^−^ was preferentially oxidized to NO_3_^−^ during NO_2_^−^ oxidation. The measured value of ^15^ε_NO2-→NO3-_ (9.2‰ ± 0.5‰ at pH 8.2 and 1 mM NO_2_^−^) was in line with previously determined ^15^ε_NO2-→NO3-_ values (9.1 to 10.2‰) of canonical *Nitrospira* NOB (11, 14) (Table 2 and Fig. S3). The NXR of *N. inopinata* clusters together with the NXR of canonical *Nitrospira* in phylogenetic analyses of the substrate-binding alpha subunit and the electron-channeling beta subunit of this enzyme (16). This close phylogenetic relationship is consistent with the highly similar kinetic isotope effects of comammox and canonical *Nitrospira*. Other NOB such as *Nitrobacter* (20.6‰) and *Nitrococcus* (12.8‰) showed remarkably stronger kinetic isotope effects than *Nitrospira* (10, 11). Interestingly, *Nitrobacter* and *Nitrococcus* have a lower whole-cell affinity (higher *K_m_*_(app)_) for NO_2_^−^ than *Nitrospira* (14, 22). Therefore, the differences in the kinetic isotope effect were suggested to be linked to the NO_2_^−^ affinity of NOB, possibly caused by a different stability of the transition state in high- versus low-affinity NXR forms (14). However, this explanation turns out to be unlikely, considering that the isotope effect of the NXR of *N. inopinata* resembles that of other *Nitrospira* strains, whereas its whole-cell nitrite affinity is low and in the same range as the whole-cell affinity of *Nitrobacter* species (21). Instead, it may be more relevant that the NXR of *Nitrospira* (including comammox species) is located in the periplasmic space (where it may interact with the cytoplasmic membrane), whereas the membrane-attached NXR of *Nitrobacter* and *Nitrococcus* is oriented toward the cytoplasm (references 26 and 27 and references cited therein). The cellular localization of NXR determines whether transport of NO_2_^−^ and NO_3_^−^ over the cytoplasmic membrane is needed, which might also influence the kinetic isotope effect through the properties of the nitrite/nitrate transporter: it may limit the expression of the isotope effect of NXR if NO_2_^−^ transport (has almost no isotope fractionation as a diffusional process) becomes limiting relative to NXR activity. The localization of NXR also affects the energy efficiency of nitrite oxidation, because only a periplasmic NXR contributes directly to proton motive force (26, 28). Moreover, the periplasmic and cytoplasmic NXR types represent phylogenetically unrelated lineages within the type II dimethyl sulfoxide (DMSO) reductase-like family of molybdopterin-containing enzymes (26, 29). The different magnitude of the kinetic isotope effect in NOB likely reflects the distinct functional properties and evolutionary history of the periplasmic and cytoplasmic NXR forms. This possibility was discussed previously (14) and gains further support from our results.

10.1128/mSphere.00634-21.3 FIG S3 Nitrite oxidation (A), isotopic signature (B) of NO_2_^−^, and ^15^ε_NO2-→NO3-_ based on substrate (ε_NXR-RS_) (C) of Nitrospira moscoviensis with 1 mM NO_2_^−^ addition. Download FIG S3, TIF file, 0.03 MB. Copyright © 2021 Liu et al. 2021 Liu et al. https://creativecommons.org/licenses/by/4.0/ This content is distributed under the terms of the Creative Commons Attribution 4.0 International license.

### Effects of substrate concentration and pH on the kinetic isotope effects of comammox bacteria.

**FIG 5:**
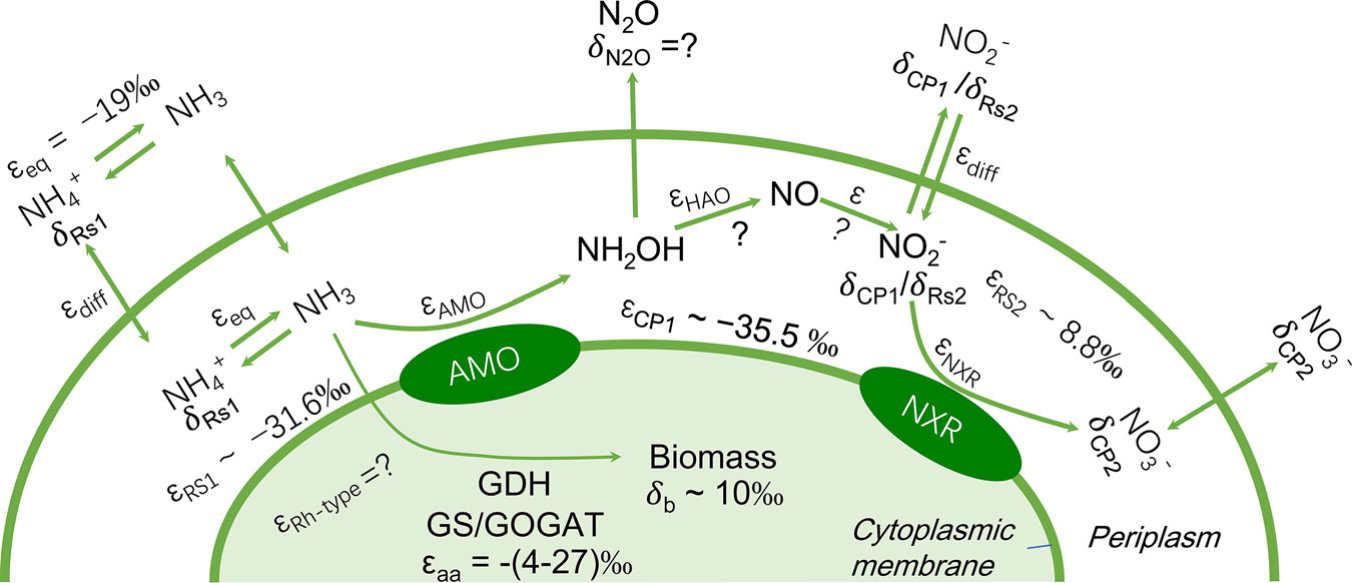
Schematic overview of N processes and isotope fractionation effects involved in NH_3_ oxidation, NO_2_^−^ oxidation, formation of intermediates, and growth of the comammox strain *Nitrospira inopinata*. Average kinetic isotope effects of NH_3_ and NO_2_^−^ oxidation are presented for the residual substrate (NH_4_^+^, ε_RS1_; NO_2_^−^, ε_RS2_) and the cumulative product (NO_2_^−^, ε_CP1_) with the addition of 1 mM NH_4_^+^ at pH 7.5. Isotope fractionation of NH_3_/NH_4_^+^ equilibration and NH_3_ uptake refer to reference 32. This schematic illustration is modified from reference 33.

Nitrogen isotope effects of ammonia oxidizers varied largely among previous studies, even within AOB and AOA (7–9). This variability might partly be caused by different enzyme (AMO) structures. However, a substantial variation in kinetic isotope effects can occur even within a single isolate, as reported for the AOB species Nitrosomonas europaea (6, 7, 30). Such variability indicates that the cultivation conditions and growth stage and specific factors, such as concentration-dependent diffusion limitations of substrate availability for the critical enzyme or the accumulation of intermediates in an N transformation pathway, can significantly affect the kinetic isotope effect (illustrated in Fig. 5). For comammox, possible effects of environmental conditions on the kinetic isotope effects of ammonia oxidation and nitrite oxidation, respectively, have not yet been studied. Here, we analyzed the effects of two conditions, the initial NH_4_^+^ concentration and culture pH, on the kinetic isotope effects of complete nitrification by *N. inopinata*.

Previous studies with AOB and AOA mostly found ^15^ε to be below −20‰, which is the range seen in our experiments with comammox at high initial NH_4_^+^ concentrations (Tables 1 and 2). However, we observed salient decreasing trends of ^15^ε_NH4+→NO2-_ with decreasing NH_4_^+^ concentrations between 1 and 0.1 mM. This result is consistent with previous data from an enrichment of thermophilic canonical AOA, where smaller isotope effects were found at lower NH_4_^+^ concentrations (0.25 mM) compared to those in experiments with high NH_4_^+^ concentrations (10 mM) (9). Also for denitrifiers, a decrease of the N isotope effect was observed along with decreasing NO_3_^−^ concentrations in the medium (31). In our study, the NH_3_ oxidation rates were lower at 0.1 mM NH_4_^+^ [2.1 μmol N (mg protein)^−1^ h^−1^] than at 0.25 mM [3.1 μmol N (mg protein)^−1^ h^−1^] and 0.5 mM NH_4_^+^ [3.8 μmol N (mg protein)^−1^ h^−1^], which were much lower than the *V*_max_ [12.8 μmol N (mg protein)^−1^ h^−1^] of *N. inopinata* (21). Thus, the rate-limiting step for NH_3_ oxidation at low NH_4_^+^ concentrations was probably more dependent on NH_3_ diffusion/transport from the extracellular space into the periplasm, where the active sites of the enzymes involved in ammonia oxidation are likely located. Therefore, under these conditions, ^15^ε_NH4+→NO2-_ did not reflect the enzymatic isotope effect, but rather the equilibrium isotope effect ^15^ε_NH4+→NH3_ at low NH_4_^+^ concentrations, which is around −19‰ (32). However, when NH_4_^+^ concentrations are higher and transport does not limit the AMO activity, enzymatically catalyzed NH_3_ oxidation will become the limiting step, and ^15^ε_NH4+→NO2-_ based on the residual substrate (ε_RS1_) will converge to the enzymatic isotope effect of AMO. Moreover, the isotope effect based on ε_RS1_ is also influenced by NH_4_^+^ assimilation for biomass formation and can therefore diverge from that based on residual substrate of ammonia oxidation. Compared to ε_RS1_, ^15^ε_NH4+→NO2-_ based on product formation (ε_CP1_) is affected by other factors, including N intermediate (NH_2_OH and NO) accumulation and N gas (N_2_O and NO) loss. Mooshammer et al. (33) demonstrated N assimilation to be the main factor responsible for the difference between ε_RS1_ and ε_CP1_ in the AOA species Nitrososphaera viennensis. In our experiment, we did not observe any significant change in the total protein content of the *N. inopinata* cultures at 0.1, 0.25, and 0.5 mM NH_4_^+^ during the incubation. The absence of detectable growth may explain the similar isotope effects of ε_RS1_ and ε_CP1_ at all three NH_4_^+^ concentrations. It appears that the NH_4_^+^ concentration also influenced the isotope effect of NO_2_^−^ oxidation, as the modeled ^15^ε_NO2-→NO3-_ was considerably smaller at the lowest tested initial NH_4_^+^ concentration (0.1 mM; Table 1). The reason could be the low maximal NO_2_^−^ concentration (around 10 μM) during NH_3_ and NO_2_^−^ oxidation with 0.1 mM NH_4_^+^ (Fig. 3A). *N. inopinata* has quite a poor affinity (372 ± 55 μM) for NO_2_^−^ during NO_2_^−^ oxidation (21), and NO_2_^−^ accumulated to higher concentrations in the experiments with 0.25 and 0.5 mM NH_4_^+^ (Fig. 3B and C). Accordingly, the kinetic isotope effect of NO_2_^−^ oxidation was more pronounced at these higher concentrations (Table 1).

Medium pH is another factor potentially affecting kinetic isotope effects. *N. inopinata* has strong activities of NH_3_ and NO_2_^−^ oxidation in the pH range of 6.5 to 8.5, with lower NH_3_ oxidation rates at pH 8.5 than those at pH 7.5 (Table 1). The NH_3_ oxidation rates were not significantly different between pH 6.5 and 7.5, while the NO_2_^−^ oxidation rates (which were calculated in the later period of NH_3_ oxidation when NH_4_^+^ was almost completely consumed) were significantly lower at pH 6.5 than that at pH 8.5. The pH also influenced the ^15^ε_NH4+→NO2-_ and ^15^ε_NO2-→NO3-._ The ε_RS1_ did not change significantly among different pH values, while the ε_CP1_ was much lower at pH 6.5 than that at pH 7.5 and pH 8.5 (Table 1). As discussed before, ε_CP1_ was probably affected by the isotopic fractionation during intermediate formation (33). With *N. inopinata* cultures, release of small amounts of NH_2_OH, NO, and N_2_O from cells has been observed during the oxidation of NH_3_ to NO_2_^−^ (34, 35). Any pH-dependent shifts in the amounts of these released compounds could lead to changes of ^15^ε_NH4+→NO2-_ (Fig. 5).

A pH-dependent shift was also observed for the ^15^ε_NO2-→NO3-_, which decreased significantly from 11.1‰ to 6.0‰ when the pH increased from 6.5 to 8.5 (Table 1). As stated above, we assume that a limited NO_2_^−^ availability caused the observed decrease of ^15^ε_NO2-→NO3-_ in the experiment with an initial NH_4_^+^ concentration of only 0.1 mM (Table 1). We observed that the concentration of transiently accumulated NO_2_^−^ was lowest at pH 8.5 (compared to pH 6.5 and 7.5) during the whole NH_3_ oxidation period (Fig. 4A to C), which was in agreement with the lower rate of NH_3_ oxidation at pH 8.5 (Table 1). In addition, the maximum NO_2_^−^ oxidation rate was higher at pH 8.5 than that at pH 6.5 and 7.5. Thus, the relatively high rate of NO_2_^−^ oxidation and low rate of NH_3_ oxidation together led to the lower NO_2_^−^ concentrations and thus can make NO_2_^−^ diffusion the limiting step for NO_2_^−^ oxidation at pH 8.5, especially for *N. inopinata* that has a low affinity for NO_2_^−^ during NO_2_^−^ oxidation. The effect of pH on NO_2_^−^ self-decomposition was unlikely the cause of the different isotope effect in the pH range of 6.5 to 8.5. In our experiments, we found no significant change of the N balance and of δ^15^N_NH3+NO2+NO3_ at pH 6.5 to 8.5 (Fig. S4). This is consistent with the findings of Casciotti et al. (10), where no change of δ^15^N was observed in the δ^15^N_NO2+NO3_ in incubations of nitrite oxidizers and in control flasks in the pH range of 7.8 to 8.8. Until now, there has been no systematic investigation of the pH effect on ^15^ε_NO2-→NO3-_ of canonical NOB. Recently, a new NOB from the genus *Nitrospira* has been cultivated from an alkaline lake (36). It would be worthy to explore the ^15^ε_NO2-→NO3-_ of alkali-tolerant NOB and the mechanisms of NO_2_^−^ oxidation at alkaline conditions in further studies.

10.1128/mSphere.00634-21.4 FIG S4 Stability of the δ^15^N_NH3+NO2+NO3_ (A) and the total nitrogen balance (B), at pH values ranging from 6.5 to 8.5, in experiment 4 (see main text). Data points show the means of three replicates (error bars depict standard deviations). Download FIG S4, TIF file, 0.04 MB. Copyright © 2021 Liu et al. 2021 Liu et al. https://creativecommons.org/licenses/by/4.0/ This content is distributed under the terms of the Creative Commons Attribution 4.0 International license.

### Conclusions.

In summary, our results demonstrate that the ^15^ε_NH4+→NO2-_ and ^15^ε_NO2-→NO3-_ of comammox *N. inopinata* ranged from −33‰ to −27‰ and 6.5‰ to 9‰, respectively, with nonlimiting NH_4_^+^ and NO_2_^−^ supply as the substrates at pH 7.5 to 8.5. Both substrate concentration and pH affected the ^15^ε_NH4+→NO2-_ and ^15^ε_NO2-→NO3-_ of *N. inopinata* during NH_3_ oxidation. At low NH_4_^+^ concentrations, especially when NH_3_ oxidation rates were much smaller than the *V*_max_ of *N. inopinata*, the ^15^ε_NH4+→NO2-_ was closer to ^15^ε_NH4+→NH3_ and did not reflect the enzymatic isotope effect of *N. inopinata*. Medium pH affected the ^15^ε_NH4+→NO2-_ of *N. inopinata* based on the cumulative product, due to the effect of pH on intermediate formation of *N. inopinata*. The exact reasons responsible for the weaker isotope effects of ^15^ε_NO2-→NO3-_ at higher pH remained elusive. Further studies should target the effects of pH and substrate concentration on the kinetic isotope effects of canonical AOB, AOA, and NOB to investigate the underlying mechanisms.

## MATERIALS AND METHODS

### Cultivation of Nitrospira inopinata.

Cultures of N. inopinata were maintained at 37°C with 1 mM NH_4_Cl in a CaCO_3_-buffered AOM medium containing (per liter) (37): 50 mg KH_2_PO4, 50 mg MgSO_4_·7H_2_O, 75 mg KCl, 584 mg NaCl, 4 g CaCO_3_ (solid buffer), 1 ml selenium-tungstate solution (SWS), and 1 ml trace element solution (TES). For the composition of TES and SWS, please refer to Widdel et al. (38). The pH of the medium was maintained at approximately 8.2. The cultivation conditions and experiments for Nitrososphaera gargensis, Nitrosocosmicus oleophilus, and Nitrospira moscoviensis are described in Text S1 in the supplemental material.

10.1128/mSphere.00634-21.6 TEXT S1 Culture conditions and description of the experiments performed with Nitrososphaera gargensis, Nitrosocosmicus oleophilus, and Nitrospira moscoviensis. Download Text S1, PDF file, 0.1 MB. Copyright © 2021 Liu et al. 2021 Liu et al. https://creativecommons.org/licenses/by/4.0/ This content is distributed under the terms of the Creative Commons Attribution 4.0 International license.

### Incubation experiments. (i) Experiment 1: ammonia oxidation with an initial concentration of 1 mM NH_4_*^+^.*

Metabolically active (i.e., ammonia-oxidizing) *N. inopinata* cells (190 ml) were harvested by centrifugation (5,000 × *g*, 30 min), washed twice with CaCO_3_-buffered medium (pH ∼8.2), and resuspended in 300 ml of CaCO_3_-buffered medium containing 1 mM NH_4_^+^. Subsequently, the cell suspension was equally distributed into three autoclaved 250-ml glass bottles. On days 0, 7, 9, 13, and 14 after inoculation, 8-ml aliquots of each replicate were transferred into 15-ml plastic tubes and centrifuged (10,000 × *g*, 10 min). Aliquots (1 ml) of the supernatant were transferred into 1-ml Eppendorf tubes for NO_2_^−^ and NO_3_^−^ isotope analysis, respectively, and 5-ml aliquots of the supernatant were transferred into 15-ml plastic tubes for NH_4_^+^ isotope analysis. All aliquots of supernatants were frozen at –20°C immediately after sampling.

### (ii) Experiment 2: nitrite oxidation with an initial concentration of 1 mM NO_2_^−^.

Metabolically active *N. inopinata* cells (2,000 ml) were harvested by centrifugation (5,000 × *g*, 30 min), washed once, and resuspended in 120 ml of CaCO_3_-buffered medium containing 1 mM NO_2_^−^. The cell suspension was equally distributed into three autoclaved 100-ml glass bottles. Samples were taken at 0, 11, 23, 35, and 47 h after inoculation and were centrifuged and stored as described above for experiment 1.

### (iii) Experiment 3: ammonia oxidation with an initial concentration of 0.1, 0.25, and 0.5 mM NH_4_^+^.

Metabolically active *N. inopinata* cells (700 ml) were harvested by centrifugation (5,000 × *g*, 30 min), washed once, and resuspended in 1,000 ml of CaCO_3_-buffered medium without NH_4_^+^. Subsequently, 900 ml of the cell suspension was equally split into nine autoclaved 250-ml glass bottles. For the different NH_4_^+^ treatments, 0.02, 0.05, and 0.1 ml of a sterile 0.5 M NH_4_^+^ solution was added to three bottles, respectively, resulting in triplicates per NH_4_^+^ concentration. Samples were taken at 0, 4, 7.5, 16.5, 22, and 28 h (0.1 mM NH_4_^+^), at 0, 7.5, 16.5, 22, 28, and 46 h (0.25 mM NH_4_^+^), and at 0, 22, 31.5, 43, 54, and 66 h (0.5 mM NH_4_^+^) after inoculation. The samples were centrifuged and stored as described above for experiment 1.

### (iv) Experiment 4: ammonia oxidation at different pH values with an initial concentration of 1 mM NH_4_^+^.

A stock culture of *N. inopinata* was transferred from CaCO_3_-buffered medium to medium buffered with 2 mM NaHCO_3_, followed by two rounds of growth and transfer in this medium, in order to remove solid CaCO_3_. Metabolically active cells (1,200 ml) were then harvested by centrifugation (8,000 × *g*, 15 min), washed with 2 mM NaHCO_3_-buffered medium, and resuspended in 10 ml of the same medium. This cell suspension was used to inoculate nine glass bottles containing 100 ml medium. During the incubations, 10 mM MES [2-(*N*-morpholino)ethanesulfonic acid], 10 mM HEPES (4-(2-hydroxyethyl)-1-piperazineethanesulfonic acid), and 10 mM TAPS ([tris(hydroxymethyl)methylamino] propanesulfonic acid) were used as buffers to adjust the pH to 6.5, 7.5, and 8.5, respectively. The MES stock buffer was prepared by dissolving 9.76 g MES in 100 ml of 136 mM NaOH. The HEPES stock buffer was prepared by dissolving 23.83 g HEPES in 100 ml of 600 mM NaOH. The TAPS stock buffer was prepared by dissolving 24.3 g TAPS in 100 ml of 570 mM NaOH. Buffer stock solutions were diluted to 10 mM MES, HEPES, and TAPS, respectively, and sterilized. 1 M HCl and 1 M NaOH were used to adjust the pH to 6.5, 7.5, and 8.5. For each pH treatment, samples were taken on 0, 0.3, 1.3, 2.3, 3.3, 4.3, 5.3, 6.5, and 8.5 days after inoculation. The samples were centrifuged and stored as described above for experiment 1.

### Chemical analyses.

Inorganic N concentrations were measured by using established protocols (39). Combined NH_3_ and NH_4_^+^ concentrations were determined by the indophenol blue method. NO_2_^−^ concentrations were measured spectrophotometrically by the Griess reaction after reaction with sulfanilamide and *N*-1-naphthyl-ethylenediamine dihydrochloride. NO_3_^−^ concentrations were measured by the Griess reaction after reduction to NO_2_^−^ with vanadium chloride. Total protein concentrations were measured by the Bradford assay.

### Nitrogen isotope analyses.

The δ^15^N values of NH_4_^+^ were analyzed by microdiffusion coupled to elemental analyzer-isotope ratio mass spectrometry (EA-IRMS [40]). For isotopic calibration, the following NH_4_^+^ standards were used: IAEA-N-2 (20.3‰ ± 0.2‰), IAEA-N-1 (0.4‰ ± 0.2‰), and USGS26 (53‰ ± 0.4‰). The δ^15^N signatures of NO_2_^−^ and NO_3_^−^ were measured by purge-and-trap isotope ratio mass spectrometry (PT-IRMS) after chemical conversion of NO_2_^−^ and NO_3_^−^ to N_2_O (40). In-house NO_2_^−^ and NO_3_^−^ standards, which ranged between −20.5 and 16.8‰, were used for isotopic calibration and were analyzed in parallel with the samples.

Natural ^15^N abundances are defined in the delta notation as follows: δ^15^N (‰) = [(^15^N_sample_/^14^N_sample_)/(^15^N_std_/^14^N_std_) − 1] × 1,000 where std stands for standard. Isotope ratios are reported relative to AIR (atmospheric dinitrogen).

Kinetic isotope effects were defined as follows:

(1) $$\varepsilon_{k}=\left(\frac{k_{H}}{k_{L}}\;-\;1\right)\times 1000$$

where *k*_L_ is the first-order rate constant for the reaction of isotopically light molecules (e.g., ^14^N) and *k_H_* is the rate constant for the reaction of isotopically heavy molecules (e.g., ^15^N). The organism-level nitrogen isotope effect for ammonia oxidation (^15^ε*_k_* ∑NH_3_/NO_2_^−^) was calculated using the Rayleigh residual substrate (RS) equation (equation 2) and the cumulative product (CP) equation (equation 3):

(2) $$\delta_{\mathrm{RS}}\;=\;\delta_{\text{input}}\;+\;\varepsilon_{k}\times \text{ ln}(1\;-\;f)$$

(3) $$\delta_{\text{CP}}=\delta_{\text{input}}\;-\;\varepsilon_{k}\text{ln}(1\;-\;f)\frac{\left(1\;-\;f\right)}{f}$$

where δ_RS_ is the N isotope signature of the residual substrate, i.e., NH_3_ (in the case of NH_3_ oxidation) or of NO_2_^−^ (in the case of NO_2_^−^ oxidation); δ_CP_ is the N isotope signature of the cumulative product, i.e., NO_2_^−^ (in the case of NH_3_ oxidation) or NO_3_^−^ (in the case of NO_2_^−^ oxidation); δ_input_ is the substrate isotope signal initially present in the medium, and *f* is the oxidizing fraction of substrate. δ^15^N at any time since the initial time point was calculated from the measured δ^15^N using the following isotope mass balance equation:

(4) $$\delta^{15}\text{N}\;=\;\frac{\left(\delta^{15}{\text{N}}_{\text{con.}}[{\text{N}}_{\text{con.}}]\;-\;\delta^{15}{\text{N}}_{\text{initial}}[{\text{N}}_{\text{initial}}]\right)}{\left([{\text{N}}_{\text{con.}}\right]\;-\;[{\text{N}}_{\text{initial}}])}$$

where N_con._ is the measured concentration of a specific substrate or product, which includes the background from initial cultures plus the added substrates, and N_initial_ is the concentration of substrates transferred with the initial cultures, i.e., the background concentrations of substrates. Initial measurements of the δ^15^N signatures of NH_4_^+^, NO_2_^−^, and NO_3_^−^, deriving from transferring the cultures to new media, when starting new experiments, were done for time zero, and corrected for from the following culture samples using mass and isotope balance equations (41).

### Solver model.

An isotope fractionation model was constructed based on linearly connected, closed system isotope fractionation submodels depicting the coupled sequential processes of nitrification. We assumed that the processes in all incubations were operating under closed system conditions as shown elsewhere (6). Three processes were considered and incorporated in the model in sequential order: (i) NH_3_ oxidation (AO), (ii) NO_2_^−^ production (NiP), and (iii) NO_2_^−^ oxidation to NO_3_^−^ (NiO). AO can also include isotope effects of equilibrium isotope fractionation between NH_3_ and NH_4_^+^ and of NH_4_^+^ uptake and is distinguished from NiP, because the kinetic isotope effect of RS and CP of NH_3_ oxidation can differ (33). The reason is that multiple processes consume NH_3_/NH_4_^+^, which is used for biomass formation and NH_3_ oxidation, and that the NH_3_ oxidation pathway in nitrifiers comprises several intermediates and by-products (hydroxylamine, nitric oxide, and nitrous oxide), which can also change the isotope effect as determined by RS and CP. The model is therefore composed of a system of nine equations (equations 5 to 13). It uses the measured N compound concentrations (NH_4_^+^_initial_, NH_4_^+^_residual_, NO_2_^−^_residual_, and NO_3_^−^_cumulative_) and their δ^15^N values (δ^15^N_NH4+ initial_, δ^15^N_NH4+ residual_, δ^15^N_NO2- residual_, and δ^15^N_NO3- cumulative_) to simulate the kinetic isotopic effects (ε) and fractions (*f*, 0 ≤ *f* ≤ 1) of N sources converted to N sinks in the three listed processes. Therefore, for the three coupled processes, we derive:

(5) $$\delta^{15}{\text{N}}_{\mathrm{NH4}\;+\;\text{residual}}=\delta^{15}{\text{N}}_{\mathrm{NH4}\;+\;\text{initial}}\;+\;\varepsilon_{\mathrm{AO}}\times \ln\left(1\;\;-\;\;f_{\text{AO}}\right)$$

(6) $$\delta^{15}{\text{N}}_{\text{NO2- cumulative}}=\delta^{15}{\text{N}}_{\mathrm{NH4}\;+\;\text{initial}}\;-\;\varepsilon_{\text{NiP}}\left(1\;-\;f_{\text{AO}}\right)\frac{\ln\left(1\;-\;f_{\text{AO}}\right)}{f_{\text{AO}}}$$

(7) $${\mathrm{NH}}_{\text{4}}^{+}{}_{\text{ residual}}={\text{NH}}_{\text{4}}^{+}{}_{\text{ initial}}\left(1\;-\;f_{\mathrm{AO}}\right)$$

(8) $${\mathrm{NO}}_{\text{2}}^{-}{}_{\text{ cumulative}}={\text{NH}}_{4}^{+}{}_{\text{ initial}}f_{\text{NiP}}$$

(9) $$\delta^{15}{\text{N}}_{\text{NO2- initial}}=\delta^{15}{\text{N}}_{\text{NO2- product}}$$

(10) $$\delta^{15}{\text{N}}_{\text{NO2-residual}}=\delta^{15}{\text{N}}_{\text{NO2-initial}}\;+\;\varepsilon_{\text{NiO}}\ln\left(1\;-\;f_{\text{NiO}}\right)$$

(11) $$\delta^{15}{\text{N}}_{\text{NO3- cumulative}}=\delta^{15}{\text{N}}_{\text{NO2-initial}}\;-{\;\varepsilon }_{\text{NiO}}\left(1\;-\;f_{\mathrm{NiO}}\right)\frac{\ln\left(1\;-\;f_{\text{NiO}}\right)}{f_{\text{NiO}}}\;$$

(12) $${\mathrm{NO}}_{2}^{-}{}_{\text{ residual}}={\text{NO}}_{2}^{-}{}_{\text{ initial}}(1\;-\;f_{\text{NiO}})$$

(13) $${\mathrm{NO}}_{3}^{-}{}_{\text{ cumulative}}={\text{NO}}_{2}^{-}{}_{\text{ initial}}f_{\text{NiO}}$$

where ε_AO_, ε_NiP_, and ε_NiO_ represent the kinetic isotopic effects of NH_3_ oxidation, NO_2_^−^ production, and NO_2_^−^ oxidation, respectively. The corresponding N fractions are denoted as *f*_AO_, *f*_NiP_, and *f*_NiO_. This system of equations was set up in Microsoft Excel and solved by the SOLVER macro in Microsoft Office Excel. The following setting was used: set objective (δ^15^N_TDN_ as measured). The variable cells (all ε and *f* values of all processes) are solved so that all modeled RS and CP values (concentrations and δ^15^N signatures) conform to the measured values. The ranges of variable cells (ε and *f* values) for each process were subject to constraints (see Table S1 in the supplemental material) with reference to published synthesis studies (7–10, 14, 33). The model was run for each incubation experiment and replicate individually, based on 1,000 iterations and using the GRG nonlinear engine as the solving method. Model accuracy across all incubation experiments was examined by regressing the simulated N contents and δ^15^N values of individual N pools against the corresponding measured values. Model results with an adjusted *R*^2^ of >0.95 were accepted. Otherwise, variable constraints were adapted and outliers were deleted eventually.

10.1128/mSphere.00634-21.5 TABLE S1 Constraints for the Solver model. Download Table S1, PDF file, 0.1 MB. Copyright © 2021 Liu et al. 2021 Liu et al. https://creativecommons.org/licenses/by/4.0/ This content is distributed under the terms of the Creative Commons Attribution 4.0 International license.

### Statistical analyses.

Analysis of variance (ANOVA) was used to test the effects of pH and concentration levels on the isotope effects of ^15^ε_NH4+→NO2-_ and ^15^ε_NO2-→NO3-_ using the R software package (version 3.4.3 [42]). Tukey’s tests (*P* < 0.05) were used to examine significant differences between the means of kinetic isotope effects at different pH or concentration levels.
