# Supplementary figures and images for: Nitrogen Kinetic Isotope Effects of Nitrification by the Complete Ammonia Oxidizer Nitrospira inopinata
Source: mSphere. 2021 Dec 8;6(6):e00634-21. doi: 10.1128/mSphere.00634-21 (PMC8653837; doi:10.1128/mSphere.00634-21)

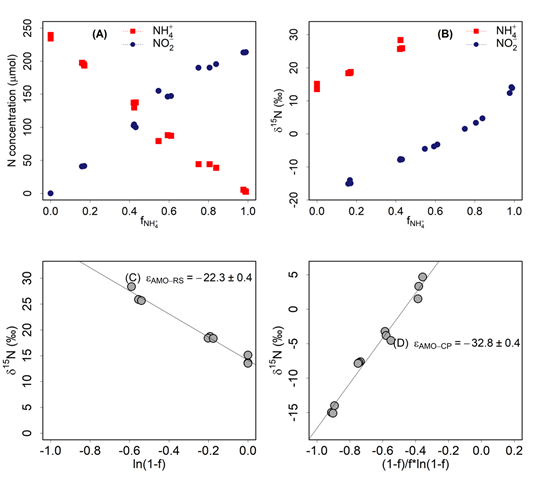

Supplement: FIG S1 [file msphere.00634-21-sf001.tif]

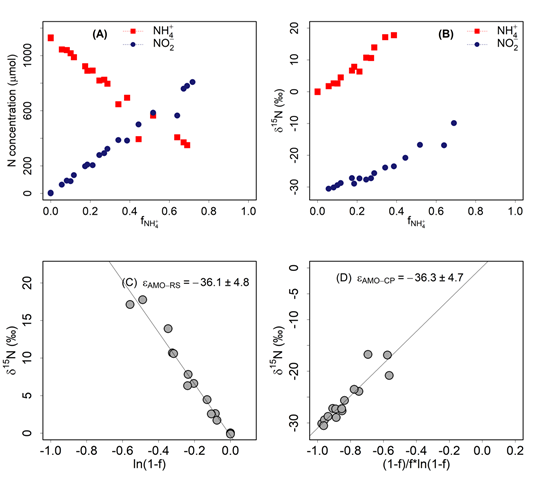

Supplement: FIG S2 [file msphere.00634-21-sf002.tif]

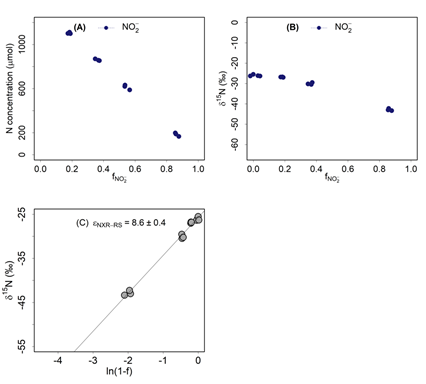

Supplement: FIG S3 [file msphere.00634-21-sf003.tif]

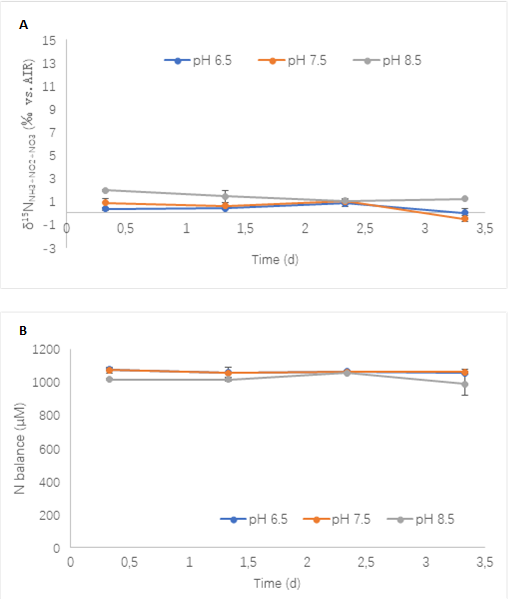

Supplement: FIG S4 [file msphere.00634-21-sf004.tif]
