# Supplementary material for: Nitrogen Kinetic Isotope Effects of Nitrification by the Complete Ammonia Oxidizer Nitrospira inopinata
Source: mSphere. 2021 Dec 8;6(6):e00634-21. doi: 10.1128/mSphere.00634-21 (PMC8653837; doi:10.1128/mSphere.00634-21)
Supplement: TEXT S1 [file msphere.00634-21-s0001.pdf]

Supplementary Text S1

In this study, we also determined nitrogen kinetic isotope effects of the two AOA species, *Nitrososphaera gargensis* and *Nitrosocosmicus oleophilus* and of the NOB *Nitrospira moscoviensis*.

Materials and Methods

General culture conditions of *Nitrososphaera gargensis*, *Nitrosocosmicus oleophilus* and *Nitrospira moscoviensis*

Cultures of *Nitrososphaera gargensis* were maintained at 37°C with 1 mM NH_4Cl in a $CaCO_3$ buffered AOM medium containing (per litre): 50 mg KH_2PO_4, 50 mg $MgSO_4 \times 7H_2O$, 75 mg KCl, 584 mg NaCl, 4 g $CaCO_3$ (solid buffer), 1 ml selenium-wolframate solution (SWS) and 1 ml trace element solution (TES). The pH of the medium was around 8.2 due to the $CaCO_3$ buffer system during the culture growth.

Cultures of *Nitrosocosmicus oleophilus* were maintained at 30°C with 1 mM NH_4Cl in an artificial freshwater (AFM) medium containing (per litre): 0.2 g KH_2PO_4, 0.4 g $MgCl_2.6H_2O$, 0.5 g KCl, 1.0 g NaCl, and 0.1 g $CaCl_2$. $2H_2O$. After autoclaving the AFM, 1 ml of TES, 1 ml of NaFeEDTA solution (7.5 mM) and 3 ml of $NaHCO_3$ (1 M) were added. The pH of the medium was adjusted to 7.0 using 1 N NaOH or HCl.

Cultures of *Nitrospira moscoviensis* were maintained at 37°C with 1 mM $NaNO_2$ in a NOB medium containing (per litre): 150 mg KH_2PO_4, 50 mg $MgSO_4 \times 7\ H_2O$, 500 mg NaCl, 10 mg $CaCO_3$, 10 mg NH_4Cl, 1ml selenium-wolframate solution (SWS) and 1ml trace element solution (TES). The pH of the medium was adjusted to 7.6-7.8.

Experiments designed for measurement of isotope fractionation in *Nitrospira moscoviensis*, *Nitrososphaera gargenis* and *Nitrosocosmicus oleophilus*

For *Nitrospira moscoviensis*, metabolically active cultures (50 ml) were harvested by centrifugation (8000g × 30 min), then washed twice, and resuspended in 300 ml fresh NOB medium with 1 mM NO_2^-. Then, the cultures were equally distributed into three autoclaved 250-ml bottles. At hours 0, 11, 23, 35 and 47, 3 ml subsamples were transferred into 15-ml falcon tubes. After centrifugation at 1000g for 10 min, 1 ml supernatant was transferred to 1-ml tubes for NO_2^- and NO_3^- isotope analysis and chemical analysis, respectively. All supernatants were frozen at -20 °C immediately after sampling.

For *Nitrososphaera gargensis*, metabolically active cultures (200 ml) were harvested by centrifugation (8000g × 30 min), then washed twice, and resuspended in 300 ml fresh $CaCO_3$ AOA/AOB medium with 0.25 mM NH_4^+. Then, the cultures were equally distributed into three autoclaved 250-ml bottles. At hours 0, 22, 43, 54, 66 and 78.5, 8 ml subsamples were transferred into 15-ml falcon tubes. After centrifugation at 1000g for 10

min, 1 ml supernatant was transferred to 1-ml tubes for NO_2^- and NO_3^- isotope analysis and chemical analysis, respectively. 5 ml supernatant was transferred to 15 ml falcon tubes for NH_4^+ isotope analysis. All supernatants were frozen at -20 °C immediately after sampling.

For *Nitrosocosmicus oleophilus*, metabolically active cultures (50 ml) were harvested by centrifugation (8000g × 30 min), then washed twice, and resuspended in 300 ml artificial fresh medium with 1 mM NH_4^+. Then, the cultures were equally distributed into three autoclaved 250-ml bottles. At days 0, 1, 2, 3, 4, 6 and 8, 8 ml subsamples were transferred into 15-ml falcon tubes. After centrifugation at 1000g for 10 min, 1 ml supernatant was transferred to 1-ml tubes for NO_2^- and NO_3^- isotope analysis and chemical analysis, respectively. 5 ml supernatant was transferred to 15 ml falcon tubes for NH_4^+ isotope analysis. All supernatants were frozen at -20 °C immediately after sampling.
