# Supplementary material for: Nitrogen Kinetic Isotope Effects of Nitrification by the Complete Ammonia Oxidizer Nitrospira inopinata
Source: mSphere. 2021 Dec 8;6(6):e00634-21. doi: 10.1128/mSphere.00634-21 (PMC8653837; doi:10.1128/mSphere.00634-21)
Supplement: TABLE S1 [file msphere.00634-21-st001.pdf]

Changing variable cells	Subject to the constraints
Isotope effect of NH_4^+ oxidized (ΔNH_4^+ ox)	$20 \leq \Delta NH_4^+ ox \leq 60$
Fractionation of oxidized NH_4^+ (f NH_4^+ ox)	$0.01 \leq f NH_4^+ ox \leq 0.999$
Isotope effect of NO_2^- production (ΔNO_2^- prod)	$10 \leq \Delta NO_2^- prod \leq 50$
Fractionation of produced NO_2^- (f NO_2^- prod)	$0.01 \leq f NO_2^- prod \leq 0.999$
Isotope effect of NO_2^- oxidized (ΔNO_2^- ox)	$-30 \leq \Delta NO_2^- ox \leq -2$
Fractionation of oxidized NO_2^- (f NO_2^- ox)	$0.01 \leq f NO_2^- ox \leq 0.999$
